# Mechanisms of Kidney Injury in Lupus Nephritis – the Role of Anti-dsDNA Antibodies

**DOI:** 10.3389/fimmu.2015.00475

**Published:** 2015-09-15

**Authors:** Susan Yung, Tak Mao Chan

**Affiliations:** ^1^Department of Medicine, Queen Mary Hospital, University of Hong Kong, Hong Kong, China

**Keywords:** lupus nephritis, anti-dsDNA antibodies, mesangial cells, proximal renal tubular epithelial cells, inflammation, fibrosis, mycophenolic acid, cyclophosphamide

## Abstract

Systemic lupus erythematosus (SLE) is an autoimmune disease characterized by a breakdown of self-tolerance, production of auto-antibodies and immune-mediated injury, resulting in damage accrual in multiple organs. Kidney involvement, termed lupus nephritis, is a major cause of morbidity and mortality that affects over half of the SLE population during the course of disease. The etiology of lupus nephritis is multifactorial and remains to be fully elucidated. Accumulating evidence suggests that in addition to forming immune complexes and triggering complement activation, anti-dsDNA antibodies contribute to the pathogenesis of lupus nephritis through binding, either directly or indirectly, to cross-reactive antigens or chromatin materials, respectively, to resident renal cells and/or extracellular matrix components, thereby triggering downstream cellular activation and proliferation as well as inflammatory and fibrotic processes. Several cross-reactive antigens that mediate anti-dsDNA antibody binding have been identified, such as annexin II and alpha-actinin. This review discusses the mechanisms through which anti-dsDNA antibodies contribute to immunopathogenesis in lupus nephritis. Corticosteroids combined with either mycophenolic acid (MPA) or cyclophosphamide is the current standard of care immunosuppressive therapy for severe lupus nephritis. This review also discusses recent data showing distinct effects of MPA and cyclophosphamide on inflammatory and fibrotic processes in resident renal cells.

## Introduction

Systemic lupus erythematosus (SLE) is a chronic autoimmune disease that affects multiple organ systems. It follows a relapsing–remitting disease course, and the risk of flare varies between individual patients. Clinical presentation can range from mild to severe depending on the affected organ. Involvement of the kidney, termed lupus nephritis, affects up to 60% of the SLE population and is more prevalent in Asians, Hispanics, Native Americans, and Blacks, especially in females of child-bearing age. Lupus nephritis is characterized by a loss of self-tolerance, production of auto-antibodies, such as those against nuclear antigens, and immune-mediated injury to the kidney. If left untreated, destruction of the normal kidney parenchyma and their replacement with fibrosis tissue will ensue. Clinical manifestations of active lupus nephritis include proteinuria, active urinary sediment, and acute renal injury.

Anti-dsDNA antibodies are specific to SLE and can be detected in patients at least 2 years before diagnosis of clinical disease (1). Serum anti-dsDNA antibody levels often reflect disease activity in lupus nephritis patients (2–5). Evidence that anti-dsDNA antibodies play an important role in disease pathogenesis originates mainly from animal studies. Intra-peritoneal administration of either human or murine anti-dsDNA antibodies to non-autoimmune mice, or their inoculation with the transgene that encodes the secreted form of an IgG anti-dsDNA antibody can induce many features of lupus nephritis (6, 7). Anti-dsDNA antibody production, their presence in immune complexes and deposition in the kidney precedes immune cell infiltration and development of proteinuria in NZBWF1 mice (8). Anti-dsDNA antibodies have also been isolated from glomeruli of lupus nephritis patients with active disease, and diverse histopathological patterns observed in lupus patients is a result of their deposition in distinct locations in the glomerulus (9).

Nephritogenic anti-dsDNA antibodies have been shown to regulate gene and protein expression of inflammatory and fibrotic mediators in resident renal cells, thereby exerting a direct effect on kidney inflammation and fibrosis (10). The precise mechanism through which anti-dsDNA antibodies are deposited in the kidney parenchyma to exert their detrimental effect remains to be fully defined, but the data to date suggest that they can either bind directly to cross-reactive antigens on the surface of resident renal cells or to components of the extracellular matrix, or indirectly through nucleosomes that are bound to constituents of the glomerular basement membrane.

This review discusses the pathogenic role of anti-dsDNA antibodies in the development of lupus nephritis, with particular focus on how they impact on inflammatory and fibrotic processes in resident kidney cells. Mechanisms through which anti-dsDNA antibodies are deposited in the kidney have been discussed elsewhere (5, 11–14).

## Detection of Anti-dsDNA Antibodies in SLE Patients

Anti-dsDNA antibodies can be detected by a variety of tests, such as the Farr radioimmunoassay (RIA), *Crithidia luciliae* indirect immunofluorescence test (CLIFT), and enzyme-linked immunosorbent assays (ELISAs). The Farr RIA and CLIFT are well-established assays that provide both diagnostic and prognostic values for SLE, whereas ELISAs are becoming more common for the measurement of anti-dsDNA antibody levels in routine clinical laboratories (15, 16).

The Farr RIA is a quantitative assay that measures the precipitation of radiolabeled dsDNA/anti-dsDNA antibody complexes. Since high salt conditions are used for precipitation, this assay preferentially detects anti-dsDNA antibodies with high avidity to dsDNA. The source of dsDNA must be carefully selected to ensure it is double-stranded, monodisperse in size with a MW >10^5^ but smaller than 10^7^ kDa to ensure reliable precipitation (17, 18). Circular double-stranded bacteriophage DNA or plasmid DNA, which can be easily iodinated after isolation are preferred (17). This assay does not distinguish between anti-dsDNA antibody Ig subclass. Disadvantages of this assay include the use of radiolabeled dsDNA, a labor-intensive methodology that cannot be automated, and detection of other proteins or compounds capable of precipitating dsDNA, thereby giving false positive results (16).

The CLIFT is a sensitive and relatively specific assay that detects anti-dsDNA antibodies with moderate to high avidity to dsDNA. It relies on indirect immunofluorescence to detect anti-dsDNA antibody binding to circular dsDNA present in the kinetoplast of *Crithidia luciliae* (19). It is noteworthy that occasional false positive results have been reported, possible due to the putative presence of histones in the kinetoplast, or lipoprotein/IgG complexes in the sample (15).

Enzyme-linked immunosorbent assays, whether “in-house” or commercial, are easy to perform, relatively inexpensive, can be automated and does not involve the use of radioisotopes. They provide quantitative results that can be readily standardized using dsDNA preparations from the World Health Organization (15). When compared to the Farr RIA and CLIFT, ELISAs have high sensitivity but less specificity as they do not distinguish between antibodies with high and low avidity to dsDNA. Discrepancies of results in independent studies have been reported and this may be due to the source and heterogeneity of the coated dsDNA, and MW and conformation of dsDNA used, the latter possibly limiting anti-dsDNA antibody interaction. False positive results may be observed if dsDNA is contaminated with single-stranded DNA or proteins, or if dsDNA coating linkers are used since they may permit binding of Ig that are not directed to dsDNA. The use of biotinylated dsDNA and coating through streptavidin to microtiter plates can reduce such errors. New ELISAs that have been optimized for the detection of anti-dsDNA antibodies of the IgG subclass with high avidity to dsDNA have been reported, and give comparable results to those obtained with the Farr RIA (20).

Anti-dsDNA antibodies can be detected in up to 80% of lupus patients suggesting that the sensitivity of current assays may not be optimal to detect low levels of anti-dsDNA antibodies, or that anti-dsDNA antibodies may be present as immune complexes in sera that prevent them from binding to dsDNA. When interpreting anti-dsDNA antibody results, clinicians should be mindful of the technique used, determine whether the assay can distinguish between high and low avidity anti-dsDNA antibodies, and note values observed in healthy controls and SLE patients with each assay.

## Origin of Pathogenic Anti-dsDNA Antibodies

Anti single-stranded DNA antibodies and anti-dsDNA antibodies constitute part of the normal repertoire of natural antibodies in healthy subjects, and are predominantly of relatively low-affinity, belong to the IgM subclass and react weakly with self-antigens (5). In SLE patients, these naturally occurring antibodies may undergo an IgM to IgG class switch or somatic mutations of the Ig-V regions to generate pathogenic anti-dsDNA antibodies. Both molecular processes are catalyzed by activation-induced deaminase (AID) in B cells within germinal centers. The importance of AID in the generation of high affinity IgG anti-dsDNA antibodies and subsequent development of lupus nephritis has been substantiated by Jiang et al. who demonstrated that lupus-prone mice deficient in AID lacked auto-reactive IgG anti-dsDNA antibodies, but produced high levels of IgM anti-dsDNA antibodies that was associated with a significant improvement in glomerulonephritis and survival compared to wild-type mice (21, 22). Class switch and somatic mutations is antigen driven and alters the specificity of B cell receptors and antibodies that they secrete, which when coupled with positive clonal selection for high antigen binding, results in the generation of high affinity antibodies (5, 23, 24). Somatic mutations where positive charged amino acids, such as arginine, asparagine, and lysine residues, are inserted within the CDRs of anti-dsDNA antibodies are critical for high affinity dsDNA binding (23, 25). Anti-dsDNA antibodies of the IgE subclass have also been implicated in the pathogenesis of lupus nephritis and are associated with active disease and increased basophil activation (26, 27).

Clearance of apoptotic bodies is defective in SLE patients and chromatin material, such as dsDNA, released from apoptotic cells would be the most likely auto-antigen to drives B cell clonal expansion. However, independent researchers have suggested that molecular mimicry may be the basis for immunological cross-reactivity (28).

## Anti-dsDNA Antibodies and Disease Pathogenicity

Lupus nephritis is initiated by the deposition of anti-dsDNA antibody-containing immune complexes in the kidney parenchyma. Notably, immune complex deposition alone is not sufficient to induce renal injury but must be accompanied by classical complement activation, infiltration of immune cells, release of chemokines, cytokines, and proteolytic enzymes, and oxidative damage, which together induce kidney inflammation and subsequent organ damage. Activation of the classical complement pathway is initiated by C1q following its binding to Ig in immune complexes. This results in a conformational change in its structure that renders it immunological. Defective clearance mechanisms results in the accumulation of C1q and predisposes SLE patients to anti-C1q antibody production (29). Genetic deficiency of C1q is rare and is associated with SLE susceptibility (30). Recent studies have shown that anti-dsDNA antibodies can cross-react with C1q (31).

Although anti-dsDNA antibody levels often correlate with disease activity, some patients can have positive or even high levels of anti-dsDNA antibodies during apparent clinical quiescence, which could indicate that not all anti-dsDNA antibodies that are detected by current assay methods are pathogenic. Factors that endow anti-dsDNA antibodies with their pathogenic potential include immunoglobulin subclasses of IgG1 and IgG3, ability to activate complement and engage Fc receptors, their charge, antigen avidity, and poly-reactivity (2, 5, 9, 32–34). For example, site-mutagenesis studies showed that substitution of aspartic acid with glycine within the heavy chain complementarity determining region of R4A, a pathogenic murine monoclonal anti-dsDNA antibody that binds to glomeruli, resulted in a loss of dsDNA binding (35). By contrast, substitution of three amino acids in R4A resulted in their deposition in renal tubules instead of glomeruli, and when this antibody was administered to SCID mice it resulted in severe proteinuria (35). It was also reported that the degree of pathogenicity of anti-dsDNA antibodies did not necessarily correlate with their affinity for dsDNA (35). These observations, together with the finding that immunization of non-autoimmune mice with mammalian DNA failed to induce nephritogenic anti-dsDNA antibody production or clinical disease manifestations would suggest that auto-reactivity to native DNA *per se* may not be an important property of pathogenic anti-dsDNA antibodies. In this context, there are data to suggest that their pathogenic nature is associated with their poly-reactivity and ability to bind directly to non-DNA cross-reactive kidney antigens (36, 37). It has been reported that antibodies directed against dsDNA and related nuclear components accounted for <10% of the total IgG eluted from kidneys from lupus nephritis patients (38).

## Binding of Anti-dsDNA Antibodies to Non-DNA Cross-Reactive Antigens in Renal Cells

Binding of anti-dsDNA antibodies to cross-reactive antigens in isolated rat kidneys was first reported by Raz et al. (39), who demonstrated that infusion of either murine monoclonal or human polyclonal anti-dsDNA antibodies alone, but not calf-thymus DNA/anti-dsDNA antibody complexes, bound to components in the glomerulus and renal interstitial blood vessels, and was accompanied by increased albumin excretion. However, the conclusion that anti-dsDNA antibodies could bind directly to cross-reactive antigens present in the kidney was subject to debate since the amount of DNA or chromatin material if any, in the anti-dsDNA antibody preparations or within the isolated kidney was not determined (39).

Many studies have focused on the interaction of anti-dsDNA antibodies with resident glomerular cells, namely mesangial and endothelial cells, and to a lesser extent on podocytes (3, 40–43). All three cell types show tri-directional communication, and thus it is not surprising that injury to one cell type will have an impact on the other cell types. The amount and location of immune deposits in the glomerulus correlate with the histopathology and severity of nephritis (9, 32). Proliferative lupus nephritis is associated with immune deposition in the mesangium and subendothelial space, whereas deposition in the subepithelial space results in membranous lesions.

In order for anti-dsDNA antibodies to mediate their detrimental effect on the kidney parenchyma, anti-dsDNA antibodies must first bind to the cell surface of resident renal cells. We and others have shown that anti-dsDNA antibodies can bind directly to annexin II, α-actinin, and ribosomal P protein in mesangial cells and trigger downstream inflammatory, apoptotic, and fibrogenic processes (28, 37, 44, 45). While anti-dsDNA antibodies have been reported to bind directly to myosin 1 and calreticulin in hepatoma cells, lymphocytes, and Chinese hamster ovary cells (46, 47), similar interactions have not been shown in resident renal cells. The mechanisms through which anti-dsDNA antibodies bind to glomerular endothelial cells and podocytes have yet to be fully characterized.

### Annexin II

Annexin II is a calcium dependent, phospholipid binding protein than can exit as a monomer, dimer, or heterotetramer in many organs, including the kidney. It is present within the cytosol of cells and translocates to the plasma membrane when the cell is activated by cytokines or growth factors. Annexin II on the surface of endothelial cells has been shown to interact with β2-glycoprotein I and toll-like receptor 4 to mediate cell activation, tissue inflammation, and thrombosis (48–51). Annexin II is also a ligand for C1q, a member of the classical complement pathway that can opsonize apoptotic cells to facilitate their phagocytic clearance (52). Defective clearance of apoptotic bodies in lupus patients is well-established, and auto-antibodies targeting annexin II have been identified in patients with proliferative lupus nephritis (37, 53) as well as in patients with rheumatoid arthritis (54).

Anti-dsDNA antibodies isolated from biopsy-proven patients with diffuse proliferative lupus nephritis can bind to annexin II on the surface of mesangial cells (37). Following their binding, anti-dsDNA antibodies are rapidly internalized and translocate to the cytosol and/or cell nucleus in a time- and temperature-dependent manner. The pathogenic nature of this interaction is highlighted by the induction of PKC activation, secretion of pro-inflammatory cytokines and hyaluronan, and matrix protein deposition (37, 55, 56). Anti-dsDNA antibodies have been reported to be able to penetrate other living cells, such as human mononuclear cells and rat H35 hepatoma cells, and the active energy-requiring internalization process was dependent on the F(ab) region (46, 57). However, Fc-receptor-mediated internalization of anti-dsDNA antibodies cannot be ruled out (58). Yanase et al. demonstrated that a proportion of anti-dsDNA antibodies can be recycled back to the cell surface of rat H35 hepatoma cells, and postulated that should anti-dsDNA antibodies be altered during the recycling process, they may also become immunogenic which could further exacerbate disease progression (59). Whether a similar mechanism occurs in mesangial cells has not been investigated. It is noteworthy that the process of antibody internalization by cells is not specific to anti-dsDNA antibodies since this process has also been observed with ribonucleoprotein, ribosomal P protein, La and Ro antibodies in non-lymphoid cells (60–62). The observation that intra-glomerular annexin II expression is increased in patients and mice with active nephritis and co-localizes with IgG and C3 deposition suggests that it may be involved in disease pathogenesis (37). Our data showed that human anti-dsDNA antibodies could induce annexin II synthesis in mesangial cells, indicating a potential amplification mechanism of immune-mediated inflammation and fibrogenesis (37). A recent report by Seret et al. showed that auto-antibodies from lupus nephritis patients targeted α-actinin and to a lesser extent laminin on the surface of kidney cells (63). That these investigators did not identify annexin II could be related to different experimental methodology and the fact that human embryonic kidney cells used in these studies have low constitutive annexin II expression (64).

### Alpha-actinin

Alpha-actinin (α-actinin) is an ubiquitous F-actin binding protein that is present in cell–cell and cell–matrix contact sites, areas of dense stress fibers and lamellipodia, which plays important roles in determining cell shape and migration (65). Nephritogenic anti-dsDNA antibodies derived from lupus-prone mice have been reported to cross-react with α-actinin, and high titers of anti-α-actinin antibodies have been detected in the serum and kidney eluates of lupus-prone mice (44). Mesangial cells of lupus-prone mice synthesize more α-actinin that cells from BALB/c mice (66). Immunization of non-autoimmune mice with α-actinin-induced anti-chromatin antibody production, glomerular Ig deposition, and proteinuria (67). These experimental data strongly suggest that α-actinin is involved in disease pathogenesis. In the clinical setting, anti-dsDNA antibodies isolated from lupus nephritis patients with active disease, but not patients with non-renal lupus, has been shown to cross-react with α-actinin (68). The association between serum immunoglobulin binding to α-actinin and disease activity in lupus nephritis patients remains controversial (36, 63, 69, 70). Adding further confusion were the findings that anti-dsDNA antibodies could bind to glomerular structures that contained extracellular nucleosomes instead of α-actinin in kidneys of NZBWF1 mice (71), and that α-actinin could not be detected in kidney eluates from nephritic lupus-prone mice (72). These discrepancies may be related to the stage and type of disease when the investigations were performed.

Although not the focus of this review, anti-dsDNA antibodies have also been shown to bind to components of the glomerular basement membrane through nucleosomes released from apoptotic cells. This interaction has been reviewed elsewhere (73, 74).

## Downstream Pathogenic Effects of Anti-dsDNA Antibodies Following their Binding to Resident Glomerular Cells

How anti-dsDNA antibodies contribute to kidney injury remains to be fully elucidated. Early studies showed that nephritogenic murine anti-dsDNA antibodies could interact directly with distinct glomerular and vascular cell surface antigens to induce mesangial expansion and proteinuria (75). Although there is evidence to show direct binding of anti-dsDNA antibodies to cross-reactive antigens in the glomerulus, it is important to note that anti-dsDNA antibody-secreting hybridomas may contain DNA and/or nucleosomes that are released into the supernatant during culture, and without prior DNase treatment these may not be removed from antibody preparations used in experiments (76). More recently, it was reported that anti-dsDNA antibodies that bound directly to basement membrane antigens showed glomerular and mesangial deposition that was not dependent on chromatin material and could activate complement and induce proteinuria in non-autoimmune mice (77).

### Cell proliferation, apoptosis, and DNase i synthesis

Interaction of nephritogenic human polyclonal anti-dsDNA antibodies with cultured human or rat mesangial cells induced cell proliferation, apoptosis, PKC activation, secretion of IL-6, IL-1β, TNF-α, TGF-β1, and hyaluronan, and fibronectin synthesis (37, 55, 56, 78, 79), suggesting that anti-dsDNA antibodies may contribute to mesangial expansion, hypercellularity, increased apoptosis, inflammation, and fibrogenesis observed in lupus nephritis. By contrast, using murine anti-dsDNA antibodies Zhang et al. observed no effect on murine mesangial cell proliferation (80).

Lupus is associated with defective clearance of apoptotic cells (81, 82). Also, anti-dsDNA antibodies have been shown to induce apoptosis in rat mesangial cells independent of changes to p53 Fas or c-myc gene expression (79). Several lines of evidence suggest that defective DNase I function may be involved in disease pathogenesis. Madaio et al. reported that murine anti-dsDNA antibodies could bind DNase I and inhibit its enzymatic activity in HL60 promyelocytic cells (83). Reports from Rekvig’s group showed that renal DNase I synthesis was suppressed by the increased intra-glomerular expression of TNF receptor-associated protein-1 in lupus-prone mice, which in turn inhibited chromatin degradation and the retention of chromatin material was associated with activation of TLR-9 and the adaptive immune system (84–86). DNase I null mice showed features present in human lupus nephritis, such as the production of auto-antibodies to chromatin, glomerular immune complex deposition, and glomerulonephritis (87). Serum DNase I activity has been reported to be decreased in patients with SLE compared to healthy individuals (87, 88).

### Glomerular inflammation

Renal inflammation and fibrosis is associated with the activation of NFκβ, MAPK, and PKC signaling pathways and induction of chemokine, cytokine, and growth factor secretion by both infiltrating and resident renal cells. Chemokine production in the glomerulus is an early event during experimental lupus nephritis and precedes cellular infiltration, proteinuria, and kidney damage (89). MCP-1 is one of the most studied chemokines in human and experimental lupus nephritis (90, 91). Anti-dsDNA antibodies can induce MCP-1 secretion in cultured mesangial cells through PKC activation and increased IL-1β secretion (92, 93). Recent studies also showed that stimulation of mesangial cells with nucleosomes could also induce MCP-1 secretion (94). Also, intra-renal expression of MCP-1 increased with disease progression in lupus mice, while MCP-1-deficient mice showed reduced inflammatory cell infiltration and proteinuria, improved kidney histology, and prolonged survival (91). Although the chemotactic properties of MCP-1 are well-established, cDNA microarray analysis of micro-dissected glomeruli from lupus nephritis patients showed that MCP-1 transcript was highly expressed in fibrosis-related gene clusters, suggesting that MCP-1 may also play an important role in the development of glomerulosclerosis (95).

Anti-dsDNA antibodies can induce IL-1β, IL-6, and TNF-α secretion in cultured mesangial and endothelial cells (37, 43, 55). IL-1β can initiate and propagate both immune and inflammatory responses in SLE through induction of itself and downstream pro-inflammatory cytokines, chemokines, hyaluronan, and adhesion molecules (55, 96–98). Although IL-1β is predominantly secreted by infiltrating macrophages, it is also locally synthesized in the kidney of lupus-prone mice and its level correlates with the severity of nephritis (97). Data from IL-1β deficient BALB/c mice demonstrated the importance of IL-1β in anti-dsDNA antibody-induced pro-inflammatory response, and IL-1β deficiency was associated with less immune complex deposition in the kidney and less proteinuria compared to wild-type mice (99). Correlation between serum IL-1β level and disease activity in SLE patients (100) further highlights the importance of IL-1β in the pathogenesis of lupus nephritis.

IL-6 is a pleotropic cytokine secreted by lymphoid and non-lymphoid cells, including resident renal cells (37, 101–106). It is a multi-functional cytokine that possesses both pro- and anti-inflammatory properties. IL-6 is critical for the differentiation and maturation of B cells and mesangial cell proliferation. In lupus nephritis, IL-6 deposition is localized to mesangial cells and podocytes and is also present alongside immune deposits in the glomerulus. Although the mechanisms through which IL-6 secretion is induced in resident renal cells has yet to be fully elucidated, we have demonstrated that binding of anti-dsDNA antibodies to annexin II in mesangial cells can induce IL-6 secretion (37). Furthermore, bidirectional communication exists between mesangial cells and human proximal renal tubular epithelial cells (PTEC) and inflammatory responses occurring in either kidney compartment induced by anti-dsDNA antibodies can provoke a response in the other compartment (104).

Similar to IL-6, TNF-α possesses both pro- and anti-inflammatory properties. In its capacity as a pro-inflammatory cytokine, TNF-α plays an important role in inflammatory processes that potentially lead to tissue damage (107). Failure to regulate TNF-α synthesis at sites of immunological injury leads to chronic activation of innate immune cells and chronic inflammatory responses (108). Serum TNF-α level and bioactivity are increased in SLE patients during flare. In the kidney, TNF-α is synthesized locally by resident renal cells and by infiltrating immune cells, and it acts synergistically with IL-1β to exacerbate intra-renal inflammatory processes. In lupus-prone mice, TNF-α is detected in glomeruli, vascular smooth muscle cells, perivascular infiltrating cells and tubular epithelial cells, and the circulating level and intra-renal expression of TNF-α correlate with proteinuria and disease activity in animal and clinical studies (109–111). Fc-receptor cross-linking on human peripheral blood monocytes induces TNF-α synthesis (112), suggesting a link between immune complex deposition and TNF-α synthesis. In addition to its role in tissue inflammation, there is evidence that TNF-α can down-regulate the adaptive immune response (108). In NZBWF1 mice, TNF-α plays a potentially protective role during the early stage of lupus nephritis since treatment with recombinant TNF-α appeared to delay the development of nephritis (113). Consistent with these findings, depletion of TNF-α accelerated the development of lupus nephritis, thus highlighting a potentially important role of TNF-α in suppressing systemic autoimmunity (114). However, in NZBWF1 mice with established nephritis, TNF-α replacement showed no beneficial effect and could even accelerate disease progression. The data suggest a biphasic role of TNF-α at different stages of disease. Possible roles of TNF-α in the pathogenesis of lupus nephritis have been reviewed (107, 115).

Using complementary DNA microarray gene profiling, Qing et al. demonstrated that incubation of mesangial cells derived from MRL/*lpr* mice with anti-dsDNA antibodies induced the pro-inflammatory genes CXCL1/KC, CX3CL1 (fractalkine), inducible nitric oxide synthase, and lipocalin 2, which are involved in neutrophil trafficking, production of nitric oxide, and transport of small lipophilic molecules (10). The induction of these pro-inflammatory mediators was facilitated in part through the binding of high-mobility group box 1 protein and engagement of TLR2 and RAGE (116).

### Mesangial fibrogenesis

If not adequately controlled, persistent inflammation induces fibrosis, a process that begins as normal wound healing response aiming to maintain or restore the normal structural and functional integrity of the kidney. Unabated inflammation causes dysregulation of the reparative processes resulting in activation of mesangial cells, endothelial-to-mesenchymal transition, infiltration of cellular mediators and increased secretion of fibrogenic chemokines, cytokines and growth factors, resulting in the over-expression and deposition of matrix proteins (117). The control of inflammation is essential to inhibit or retard progressive kidney fibrosis. In immune-mediated kidney diseases, accumulation of extracellular matrix proteins in the glomerulus results in the development of glomerulosclerosis. Although tubulointerstitial changes better predict long-term renal prognosis (118), Vleming et al. reported that the extent of intra-glomerular fibronectin staining correlated with structural abnormalities in the glomerulus and severity of renal insufficiency (119).

Fibronectin is a large glycoprotein that is essential for renal homeostasis and resident renal cell proliferation, adhesion, migration, differentiation, and survival. In the normal kidney, fibronectin expression is limited to the Bowman’s capsule, mesangial matrix, and glomerular basement membrane where it interacts with other matrix proteins to maintain the structural integrity of the glomerular capillary. We and others have demonstrated that intra-glomerular fibronectin expression is increased in patients and mice with active lupus nephritis (56, 120, 121), and its co-localization with IgG deposition suggests an association between auto-antibody deposition and matrix protein accumulation (56). The underlying mechanism that initiates increased intra-glomerular fibronectin synthesis in lupus nephritis remains to be fully elucidated. We have shown that anti-dsDNA antibodies induced rapid and sustained phosphorylation of PKC-α, PKC-βI, and PKC-βII in cultured mesangial cells, which resulted in increased TGF-β1 bio-activation and subsequent fibronectin synthesis and deposition in the extracellular milieu (56). In line with our findings, Zhang et al. also reported that anti-dsDNA antibodies could induce mediators of fibrosis in mesangial cells (80). The role of TGF-β1 in kidney fibrosis is well-established (122, 123). The importance of PKC-β in the development of lupus nephritis has been highlighted by Oleksyn et al., who demonstrated that PKC-β deficiency abrogated auto-antibody production, proteinuria, and histological features of kidney disease in lupus-prone mice (124).

## Binding of Anti-dsDNA Antibodies to Proximal Renal Tubular Epithelial Cells and their Induction of Inflammatory and Fibrotic Processes

Although lupus nephritis is likely to be initiated in the glomerulus, injury is rarely contained in this compartment and often extends into the tubulo-interstitium. The degree of tubular injury and interstitial fibrosis rather than glomerulosclerosis correlates with progression of renal insufficiency (118). We and others have reported that immune deposits along the tubular basement membrane occurred in up to 70% of patients with diffuse proliferative lupus nephritis. The amount of immune complexes deposited in the tubulointerstitium correlates with circulating anti-dsDNA antibodies, tubulointerstitial inflammatory cell infiltration, IL-6 expression, tubular atrophy, and interstitial fibrosis (104). While tubulointerstitial injury heralds poor long-term kidney prognosis in lupus nephritis patients, the mechanisms leading to tubulointerstitial damage is poorly understood. Anti-dsDNA antibodies can induce epithelial-to-mesenchymal transition (EMT) in cultured PTEC, a process that precedes kidney fibrosis and is characterized by a loss of epithelial markers and replacement of their cobblestone, epithelial morphology by an elongated, fibroblastic appearance, with concomitant *de novo* synthesis of mesenchymal markers, such as α-smooth muscle actin, β-catenin, SNAIL, fibronectin, and collagen I. EMT markers are increased in renal biopsies from lupus nephritis patients and their expression is associated with renal impairment, interstitial leukocyte infiltration, and tubulointerstitial fibrosis (125, 126).

Anti-dsDNA antibodies can also induce cell-associated and soluble fibronectin synthesis in PTEC through activation of ERK, JNK, p38, PKC-α, and PKC-βII, and subsequent induction of TGF-β1, IL-6, IL-8, MCP-1, and TNF-α secretion (127). It is noteworthy that human anti-dsDNA antibodies can induce secretion of pro-inflammatory cytokines in PTEC, suggesting that even during apparent clinical quiescence, ongoing subclinical inflammation may occur in the kidney. Consistent with our findings, Ronda et al. demonstrated that serum IgG from SLE patients-induced IL-6 secretion in PTEC through ERK activation (128). The ability of anti-dsDNA antibodies to increase both cell-associated and soluble fibronectin suggests that these auto-antibodies can induce tubulointerstitial fibrosis through two distinct mechanisms. While cell-associated fibronectin will deposit in the extracellular matrix and contribute to matrix protein accumulation, soluble fibronectin may amplify fibrotic processes through increased TGF-β1 secretion and induction of collagen I synthesis (127). The observation that the aforementioned pro-inflammatory cytokines can also induce fibronectin synthesis (127) suggests that these peptides not only contribute to kidney inflammation but also to tubulointerstitial fibrosis (127). It is thus possible that these chemokines and cytokines act synergistically with each other or with fibrotic growth factors to facilitate the recruitment of myofibroblasts and other effector cells to sites of injury with mutual signaling pathways involved in both kidney inflammation and fibrosis during lupus nephritis.

How anti-dsDNA antibodies bind to PTEC is currently unknown although our preliminary studies suggest that it is possibly through cross-reactive antigens. Monoclonal murine anti-dsDNA antibodies have been shown to bind PK15 cells, a porcine proximal tubular epithelial cell line, through A and D SnRNP proteins, which mediate their internalization and compartmentalization either in the cytosol or nucleus resulting in modest cell lysis (129). Another sub-set of murine anti-dsDNA antibodies bind PK15 cells, are not internalized and induce significant cell lysis, especially in the presence of complement (129). Whether these observations are relevant to human PTEC remains to be studied, but with regard to cytotoxicity, human polyclonal anti-dsDNA antibodies can induce cell detachment and decrease human PTEC viability, thereby confirming their pathogenic nature (104).

## Effects of Mycophenolic Acid and Cyclophosphamide on Inflammatory and Fibrotic Processes Induced by Anti-dsDNA Antibodies

Prevention of fibrosis and preservation of normal kidney histology are essential to ensure long-term renal and patient survival (130). Standard treatment for active lupus nephritis entails the use of corticosteroids combined with an immunosuppressive agent, such as mycophenolate mofetil (MMF) or cyclophosphamide. Although MMF and cyclophosphamide show comparable treatment efficacy, MMF is associated with less side effects (131). Mycophenolic acid (MPA), the active metabolite of MMF, exerts its therapeutic effect through inhibition of lymphocyte proliferation, especially those that are activated (132). We and others have demonstrated that MPA can exert anti-proliferative, anti-inflammatory, and anti-fibrotic effects on non-immune cells that are independent of its immunosuppressive actions (56, 103, 121, 127, 133–135). At clinically relevant doses, MPA significantly reduced anti-dsDNA antibody-induced cell proliferation and fibrogenic processes through the inhibition of PKC activation in human mesangial cells (56). Similarly, MPA inhibited anti-dsDNA antibody-mediated activation of the MAPK and PKC signaling pathways in PTEC, with a concomitant decrease in synthesis of cell-associated and soluble fibronectin, and secretion of TGF-β1, IL-6, IL-8, and TNF-α (127). When compared to cyclophosphamide, MPA was more effective in suppressing anti-dsDNA antibody-induced PKC-α activation, TGF-β1 secretion and fibronectin production (121). Furthermore, MPA and cyclophosphamide could both decrease TGF-β1 and TNF-α-induced fibronectin synthesis in human mesangial cells, whereas IL-6-induced fibronectin was suppressed only by MPA (121). In experimental studies, MMF together with methylprednisolone was more effective than cyclophosphamide and methylprednisolone in suppressing glomerulosclerosis in lupus-prone mice (121). The overall results suggest that anti-dsDNA antibodies contribute to matrix deposition and glomerulosclerosis, and that MPA treatment is associated with more anti-fibrotic effect when compared with cyclophosphamide, thereby may be more beneficial in preventing progression to chronic kidney failure.

## Conclusion

Lupus nephritis is characterized by glomerular and tubulointerstitial inflammation, which could lead to progressive glomerulosclerosis and tubulointerstitial fibrosis if not interrupted. How anti-dsDNA antibodies bind to resident renal cells and components of the glomerular basement membrane to trigger downstream kidney inflammation and fibrosis is a topic of much interest and debate, and it is likely that we have only uncovered the tip of the iceberg. Many chemokines, cytokines, and growth factors have multi-faceted roles during lupus nephritis, which could complicate how we perceive the role of each molecule. Inflammatory and fibrotic processes are often intercalated and bidirectional signaling between different compartments of the kidney appears a critical factor to amplify injurious responses. Deciphering how these peptides contribute not only in the initial pathogenic events but also in the effector phase to mediate kidney damage is essential before suitable therapeutic strategies can be devised.

**Figure 1:**
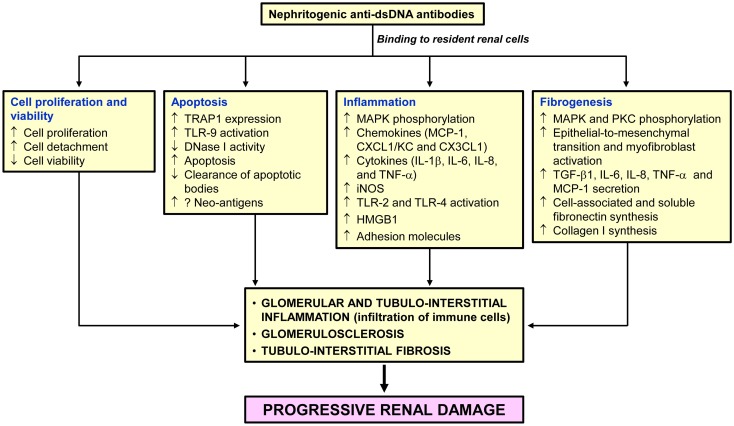
**Pathogenic changes in the kidney following anti-dsDNA antibody binding to resident renal cells**. Binding of pathogenic anti-dsDNA antibodies to resident glomerular and tubulointerstitial renal cells contributes to cell proliferation, and inflammatory, apoptotic and fibrogenic processes. If not adequately controlled, destruction of the normal kidney parenchyma and their replacement with fibrosis tissue will follow leading to end-stage renal disease.

Figure 1 summarizes the putative pathogenic changes that may occur in the kidney following anti-dsDNA antibody binding to resident renal cells.

## Conflict of Interest Statement

The authors declare that the research was conducted in the absence of any commercial or financial relationships that could be construed as a potential conflict of interest.

## Abbreviations

AID, activation-induced deaminase; C1q, complement 1q; CLIFT, *Crithidia luciliae* indirect immunofluorescence test; CXCL1, (C–X–C motif) ligand 1; dsDNA, double-stranded DNA; ELISA, enzyme-link immunosorbent assay; EMT, epithelial-to-mesenchymal transition; ERK, extracellular signal-regulated kinase; HMGB1; high-mobility group box 1; IgG, immunoglobulin G; IL-1β, interleukin-1β; IL-6, interleukin-6; IL-8, interleukin-8; iNOS, inducible nitric oxide synthase; JNK, jun N-terminal kinase; MAPK, mitogen-activated protein kinase; MCP-1, monocyte chemoattractant protein-1; MMF, mycophenolate mofetil; MPA, mycophenolic acid; MW, molecular weight; NZBWF1, New Zealand Black and White F1 generation; PKC, protein kinase C; RAGE, receptor for advanced glycation end-products; SLE, systemic lupus erythematosus; TLR, toll-like receptor, TGF-β1, transforming growth factor-β1; TNF-α, tumor necrosis factor-α; TRAP1, TNF receptor-associated protein-1.
